# Constructive techniques for zeros of monotone mappings in certain Banach spaces

**DOI:** 10.1186/s40064-015-1169-2

**Published:** 2015-07-28

**Authors:** C Diop, T M M Sow, N Djitte, C E Chidume

**Affiliations:** Gaston Berger University, Saint Louis, Senegal; Department of Mathematics, African University of Sciences and Technology, Abuja, Nigeria

**Keywords:** Strongly monotone, Lipschitz, Bounded

## Abstract

Let *E* be a 2-uniformly convex real Banach space with uniformly Gâteaux differentiable norm, and $E^*$ its dual space. Let $A:E\rightarrow E^*$ be a bounded strongly monotone mapping such that $A^{-1}(0)\ne \emptyset .$ For given $x_1\in E,$ let $\{x_n\}$ be generated by the algorithm:

$$\begin{aligned} x_{n+1}= J^{-1}( Jx_n -\alpha _nAx_n),\,n\ge 1, \end{aligned}$$

where *J* is the normalized duality mapping from *E* into $E^*$ and $\{\alpha _n\}$ is a real sequence in (0, 1) satisfying suitable conditions. Then it is proved that $\{x_n\}$ converges strongly to the unique point $x^*\in A^{-1}(0).$ Finally, our theorems are applied to the convex minimization problem.

## Background

Let *H* be a real Hilbert space with inner product $\langle \cdot ,\cdot \rangle _H$ and norm $\Vert \cdot \Vert _H.$ An operator $A:H\rightarrow H$ is called *monotone* if

1.1 $$\begin{aligned} \langle Ax-Ay,x-y\rangle _H \ge 0\,\,\forall \,x,y\in H, \end{aligned}$$

and is called *strongly monotone* if there exists $k\in (0,1)$ such that

1.2 $$\begin{aligned} \langle Ax-Ay,x-y\rangle _H \ge k\Vert x-y\Vert ^2_H \,\,\forall x,y\in H. \end{aligned}$$

Interest in monotone operators stems mainly from their usefulness in numerous applications. Consider, for example, the following: Let $f:H\rightarrow \mathbb {R}\cup \{\infty \}$ be a proper lower semi continuous and convex function. The *subdifferential*, $\partial f:H\rightarrow 2^{H}$ of *f* at $x\in H$ is defined by

$$\begin{aligned} \partial f(x)=\big \{x^*\in H:f(y)-f(x)\ge \langle y-x,x^*\rangle \,\,\forall \,y\in H\big \}. \end{aligned}$$

It is easy to check that $\partial f:H\rightarrow 2^H$ is a *monotone operator* on *H*, and that $0\in \partial f(x)$ if and only if *x* is a minimizer of *f*. Setting $\partial f\equiv A,$ it follows that solving the inclusion $0\in Au,$ in this case, is solving for a minimizer of *f*.

Several existence theorems have been established for the equation $Au=0$ when *A* is of the monotone-type (see e.g., Deimling 1985; Pascali and Sburian 1978).

The extension of the monotonicity definition to operators from a Banach space into its dual has been the starting point for the development of nonlinear functional analysis. The monotone maps constitute the most manageable class because of the very simple structure of the monotonicity condition. The monotone mappings appear in a rather wide variety of contexts since they can be found in many functional equations. Many of them appear also in calculus of variations as subdifferential of convex functions. (Pascali and Sburian 1978, p. 101).

Let *E* be a real normed space, $E^*$ its topological dual space. The map $J:E\rightarrow 2^{E^*}$ defined by

$$\begin{aligned} Jx:=\big \{x^*\in E^*:\langle x,x^*\rangle =\Vert x\Vert .\Vert x^*\Vert ,\,\Vert x\Vert =\Vert x^*\Vert \big \} \end{aligned}$$

is called the *normalized duality map* on *E*. where, $\langle ,\rangle$ denotes the generalized duality pairing between *E* and $E^*.$

A map $A:E\rightarrow E^{*}$ is called *monotone* if for each $x,y\in E,$ the following inequality holds:

1.3 $$\begin{aligned} \big \langle Ax-Ay,x-y\big \rangle \ge 0. \end{aligned}$$

*A* is called *strongly monotone* if there exists $k\in (0,1)$ such that for each $x,y\in E,$ the following inequality holds:

1.4 $$\begin{aligned} \langle Ax-Ay,x-y\rangle \ge k\Vert x-y\Vert ^2. \end{aligned}$$

A map $A:E\rightarrow E$ is called *accretive* if for each $x,y\in E,$ there exists $j(x-y)\in J(x-y)$ such that

1.5 $$\begin{aligned} \big \langle Ax-Ay,j(x-y)\big \rangle \ge 0. \end{aligned}$$

*A* is called *strongly accretive * if there exists $k\in (0,1)$ such that for each $x,y\in E,$ there exists $j(x-y)\in J(x-y)$ such that

1.6 $$\begin{aligned} \langle Ax-Ay,j(x-y)\rangle \ge k\Vert x-y\Vert ^2. \end{aligned}$$

In a Hilbert space, the normalized duality map is the identity map. Hence, in Hilbert spaces, *monotonicity* and *accretivity* coincide. For accretive-type operator *A*, solutions of the equation $Au=0,$ in many cases, represent *equilibrium state* of some dynamical system (see e.g., Chidume 2009, p. 116).

For approximating a solution of $Au=0,$ assuming existence, where $A:E\rightarrow E$ is of accretive-type, Browder (1967) defined an operator $T:E \rightarrow E$ by $T:=I-A,$ where *I* is the identity map on *E*. He called such an operator *pseudo-contractive*. It is trivial to observe that zeros of *A* correspond to fixed points of *T*. For Lipschitz strongly pseudo-contractive maps, Chidume (1987) proved the following theorem.

### **Theorem C1**

(Chidume 1987)* Let*$E =L_{p}, \,2\le p < \infty,$* and*$K\subset E$* be nonempty closed convex and bounded. Let*$T:K\rightarrow K$* be a strongly pseudo-contractive and Lipschitz map. For arbitrary*$x_{0}\in K,$* let a sequence*$\{x_{n}\}$* be defined iteratively by*$x_{n+1} = (1-\lambda _{n})x_{n} + \lambda _{n} Tx_{n},\,n\ge 0,$* where*$\{\lambda _{n}\} \subset (0,1)$* satisfies the following conditions:*$(i)\,\sum _{n=1}^{\infty }\lambda _{n}= \infty , \,\,(ii)\sum _{n=1}^{\infty }\lambda _{n}^{2} < \infty.$* Then,*$\{x_{n}\}$* converges strongly to the unique fixed point ofT*.

By setting $T:=I-A$ in Theorem C1, the following theorem for approximating a solution of $Au=0$ where *A* is a strongly accretive and bounded operator can be proved.

### **Theorem C2**

*Let*$E =L_{p}, \,2\le p < \infty.$* Let*$A:E\rightarrow E$* be a strongly accretive and bounded map. Assume*$A^{-1}(0)\ne \emptyset .$* For arbitrary*$x_{0}\in K,$* let a sequence*$\{x_{n}\}$* be defined iteratively by*$x_{n+1} =x_{n} - \lambda _{n} Ax_{n},\,n\ge 0,$* where*$\{\lambda _{n}\} \subset (0,1)$* satisfies the following conditions:*$(i)\,\sum _{n=1}^{\infty }\lambda _{n}= \infty , \,\,(ii)\sum _{n=1}^{\infty }\lambda _{n}^{2} < \infty.$* Then,*$\{x_{n}\}$* converges strongly to the unique solution of*$Au=0.$

The main tool used in the proof of Theorem C1 is an inequality of Bynum (1976). This theorem signalled the return to extensive research efforts on inequalities in Banach spaces and their applications to iterative methods for solutions of nonlinear equations. Consequently, Theorem C1 has been generalized and extended in various directions, leading to flourishing areas of research, for the past thirty years or so, for numerous authors (see e.g., Censor and Reich 1996; Chidume 1986, 1987, 2002; Chidume and Bashir 2007; Chidume and Chidume 2005, 2006; Chidume and Osilike 1999; Deng 1993; Zhou and Jia 1996; Liou 1990; Qihou 1990; Reich 1977, 1978, 1979; Reich and Sabach 2009, 2010; Weng 1991; Xiao 1998; Xu 1991, 1991, 1992; Berinde and Maruster 2014; Moudafi 2003, 2004; 2010; Moudafi and Thera 1997; Xu and Roach 1991; Xu et al. 1995; Zhu 1994 and a host of other authors). Recent monographs emanating from these researches include those by Berinde (2007), Chidume (2009), Goebel and Reich (1984), and William and Shahzad (2014).

Unfortunately, the success achieved in using geometric properties developed from the mid 1980s to early 1990s in approximating zeros of *accretive-type mappings* has not carried over to approximating zeros of *monotone-type operators* in general Banach spaces. Part of the problem is that since *A* maps *E* to $$E^{*},$$ for $$x_{n}\in E,$$$$Ax_{n}$$ is in $$E^{*}.$$ Consequently, a recursion formula containing $$x_{n}$$ and $$Ax_{n}$$ may not be well defined.

Attempts have been made to overcome this difficulty by introducing the inverse of the normalized duality mapping in the recursion formulas for approximating zeros of monotone-type mappings.

In this paper, we introduce an *iterative scheme of Mann-type* to approximate the unique zero of *a strongly monotone bounded mapping* in 2-uniformly convex real Banach with uniformly Gâteaux differentiable norm. Then we apply our results to the convex minimization problem. Finally, our method of proof is of independent interest.

### *Remark 1*

In $$L_{p}$$ spaces, $$1<p<\infty, $$ the formula for *J* is known precisely (see e.g., Chidume 2009; Cioranescu 1990). In fact, from Cioranescu (1990), Corollary 4.10, p. 72, we have for $$J:L_p\rightarrow {L_p}^*, $$$$1<p<\infty, $$

$$\begin{aligned} J(f)=|f|^{p-1}\cdot sign\frac{f}{\Vert f\Vert ^{p-1}}. \end{aligned}$$

## Preliminaries

Let *E* be a normed linear space. *E* is said to be smooth if

2.1 $$\begin{aligned} \lim \limits _{t\rightarrow 0}\frac{\Vert x+ty\Vert - \Vert x\Vert }{t} \end{aligned}$$

exist for each $x,y \in S_E$ (Here $S_E:=\{x\in E : ||x||=1\}$ is the unit sphere of *E*). *E* is said to be uniformly smooth if it is smooth and the limit is attained uniformly for each $x,y\in S_E,$ and *E* is Fréchet differentiable if it is smooth and the limit is attained uniformly for $y\in S_E.$

A normed linear space *E* is said to be strictly convex if:

$$\begin{aligned} \Vert x\Vert =\Vert y\Vert =1,\,\,x\ne y\,\,\Rightarrow \,\,\,\Big \Vert \frac{x+y}{2}\Big \Vert <1. \end{aligned}$$

The modulus of convexity of *E* is the function $\delta _E: (0,2]\rightarrow [0,1]$ defined by:

$$\begin{aligned} \delta _E(\epsilon ):= \inf \Big \{1-\frac{1}{2}\Vert x+y\Vert \,\,:\Vert x\Vert =\Vert y\Vert =1,\,\Vert x-y\Vert \ge \epsilon \Big \}. \end{aligned}$$

*E* is uniformly convex if and only if $\delta _E(\epsilon )>0$ for every $\epsilon \in (0,2].$ Let $p>1.$ Then *E* is said to be *p*-uniformly convex if there exists a constant $c>0$ such that $\delta _E(\epsilon )\ge c\epsilon ^p$ for all $\epsilon \in (0,2].$ Observe that every *p*-uniformly convex space is uniformly convex.

It is well known that *E* is smooth if and only if *J* is single valued. Moreover, if *E* is a reflexive smooth and strictly convex Banach space, then $J^{-1}$ is single valued, one-to-one, surjective and it is the duality mapping from $E^*$ into *E*. Finally, if *E* has uniform Gâteaux differentiable norm, then *J* is norm-to-weak$^*$ uniformly continuous on bounded sets.

In the sequel, we shall need the following results and definitions.

### **Theorem 2.1**

(Xu 1991)* Let*$p>1$* be a given real number. Then the following are equivalent in a Banach space:*

(i) *E isp*-*uniformly convex*.
(ii) *There is a constant*$c_1>0$* such that for every*$x,y\in \,E$* and*$j_x\in \,J_p(x),$* The following inequality holds:*
  $$\begin{aligned} \Vert x+y\Vert ^p\ge \Vert x\Vert ^p + p\langle y,j_x\rangle + c_1\Vert y\Vert ^p. \end{aligned}$$
(iii) *There is a constant*$c_2>0$* such that for every*$x,y\in \,E$* and*$j_x\in \,J_p(x), j_y\in \,J_p(y),$* the following inequality holds:*
  $$\begin{aligned} \langle x-y,j_x-j_y\rangle \ge c_2\Vert x-y\Vert ^p. \end{aligned}$$

### **Corollary 2.2**

*LetE be a 2-uniformly convex and smooth real Banach space. Then*$J^{-1}$* is Lipschtzian form*$E^*$* intoE, i.e., there exists constant*$L>0$* such for all*$u,v\in E^*$* the following holds*

2.2 $$\begin{aligned} \Vert J^{-1}u-J^{-1}v\Vert \le L||u-v\Vert . \end{aligned}$$

### *Proof*

This follows from inequality (*iii*) of Theorem 2.1 with $$p=2.$$$$\square $$

Let *E* be a smooth real Banach space with dual $E^*.$ The function $\phi :E\times E \rightarrow \mathbb {R},$ defined by

2.3 $$\begin{aligned} \phi (x,y)=\Vert x\Vert ^2-2\langle x,Jy\rangle +\Vert y\Vert ^2, \,\, x,y \in E, \end{aligned}$$

where *J* is the normalized duality mapping from *E* into $E^*,$ introduced by Alber has been studied by Alber (1996), Alber and Guerre-Delabiere (2001), Kamimura and Takahashi (2002), Reich (1979) and a host of other authors. This functional $\phi$ will play a central role in what follows. If $E=H,$ a real Hilbert space, then Eq. (2.3) reduce to $\phi (x,y)=\Vert x-y\Vert ^2$ for $x,y\in H.$ It is obvious from the definition of the function $\phi$ that

2.4 $$\begin{aligned} (\Vert x\Vert -\Vert y\Vert )^2\le \phi (x,y)\le (\Vert x\Vert +\Vert y\Vert )^2 \,\,\forall \, x,y\in E. \end{aligned}$$

Define a functional $V:E\times E^* \rightarrow \mathbb {R}$ by

2.5 $$\begin{aligned} V(x,x^*)=\Vert x\Vert ^2-2\langle x,x^*\rangle +\Vert x^*\Vert ^2, \,\, x\in E, x^*\in E^*. \end{aligned}$$

Then, it is easy to see that

2.6 $$\begin{aligned} V(x,x^*)=\phi (x,J^{-1}x^*)\,\, \forall \, x\in E,\,x^* \in E^*. \end{aligned}$$

### **Lemma 2.3**

(Alber 1996)* LetE be a reflexive strictly convex and smooth real Banach space with*$E^*$* as its dual. Then,*

2.7 $$\begin{aligned} V(x,x^*)+2\langle J^{-1}x^*-x, y^*\rangle \le V(x,x^*+y^*) \end{aligned}$$

*for all*$x\in E$* and*$x^*,y^* \in E^*.$

### **Lemma 2.4**

(Kamimura and Takahashi 2002)* LetE be a smooth uniformly convex real Banach space, and let*$\{x_n\}$* and*$\{y_n\}$* be two sequences ofE*.* If either*$\{x_n\}$* or*$\{y_n\}$* is bounded and*$\phi (x_n,y_n)\rightarrow 0$* as*$n\rightarrow \infty,$* then*$\Vert x_n-y_n\Vert \rightarrow 0$* as*$n\rightarrow \infty .$

### **Lemma 2.5**

(Tan and Xu 1993)* Let*$\{a_n\}$* be a sequence of non-negative real numbers satisfying the following relation:*

$$\begin{aligned} a_{n+1}\le a_n + \sigma _n\,\, n\ge 0. \end{aligned}$$

*Such that*$\displaystyle \sum _{n=0}^\infty \sigma _n< \infty .$* Then*$\displaystyle \lim _{n\rightarrow \infty }a_n$* exists. If addition, the sequence*$\{a_n\}$* has a subsequence that converges to 0. Then*$\{a_n\}$* converges to 0.*

The following results will be useful.

### **Lemma 2.6**

(Alber and Ryazantseva 2006)* For*$p>1,$* letX be ap*-*uniformly convex and smooth real Banach space andS a bounded subset ofX*.* Then there exists a positive constant*$\alpha$* such that*

$$\begin{aligned} \alpha \Vert x-y\Vert ^p\le \phi (x,y)\,\,\forall \,x,y\in S. \end{aligned}$$

### **Lemma 2.7**

*LetE be a 2-uniformly convex smooth real Banach space. Then the following inequality holds:*

$$\begin{aligned} ||x-y\Vert ^2\ge \phi (x,y)+(c_1-1)\Vert x\Vert ^2\,\, x,y\in E, \end{aligned}$$

*where*$0\le c_1\le 1$* has the same meaning as in Theorem*2.1.

### *Proof*

Using (*ii*) of Theorem 2.1, we have

$$\begin{aligned} \Vert x-y\Vert ^2\ge \Vert x\Vert ^2 - 2\langle y,Jx\rangle + c_1\Vert y\Vert ^2. \end{aligned}$$

Interchanging *x* and *y*, we obtain

$$\begin{aligned} \Vert x-y\Vert ^2&\ge \Vert y\Vert ^2 -2\langle x,Jy\rangle + c_1\Vert x\Vert ^2\\&= \phi (x,y) + (c_1-1)\Vert x\Vert ^2. \end{aligned}$$

$$\square $$

## Main results

We now prove the following result

### **Theorem 3.1**

*LetE be a 2-uniformly convex real Banach space with uniformly Gâteaux differentiable norm and*$E^*$* its dual space. Let*$A:E\rightarrow E^*$* be a bounded andk*-*strongly monotone mapping such that*$A^{-1}(0)\ne \emptyset .$* For arbitrary*$x_1\in E,$* let*$\{x_n\}$* be the sequence defined iteratively by:*

3.1 $$\begin{aligned} x_{n+1}=J^{-1}(Jx_n-\alpha _nAx_n),\,\, n\ge 1, \end{aligned}$$

*whereJ is the normalized duality mapping fromE into*$$E^*$$* and*$$\{\alpha _n\}\subset (0,1)$$* is a real sequence satisfying the following conditions:*$$(i)\,\,\sum _{n=1}^\infty \alpha _n=\infty; $$$$(ii)\,\, \sum _{n=0}^\infty \alpha _n^{2}< \infty .$$* Then, there exists*$$\gamma _0>0$$* such that if*$$\alpha _n<\gamma _0,$$* the sequence*$$\{x_n\}$$* converges strongly to the unique solution of the equation*$$Au=0.$$

### *Proof*

The proof is in two steps:

**Step 1:** We prove that $\{x_n\}$ is bounded. Since $A^{-1}(0)\ne \emptyset,$ let $x^*\in A^{-1}(0).$There exists $r>0$ such that:

3.2 $$\begin{aligned} r\ge \max \Big \{{4(1- c_1)\Vert x^*\Vert ^2,\phi (x_1,x^*)} \Big \}. \end{aligned}$$

We show that $\phi (x_n,x^*)\le r$ for all $n\ge 1.$ The proof is by induction. We have $\phi (x_1,x^*)\le r.$ Assume that $\phi (x_n,x^*)\le r$ for some $n\ge 1.$ We show that $\phi (x_{n+1},x^*)\le r.$ From the induction assumption and Lemma 2.6, there exists $\alpha ^*>0$ such that $\Vert x_n-x^*\Vert ^2\le r\alpha ^*.$ Since *A* is bounded, we have:

3.3 $$\begin{aligned} M_0=2L\sup \{\Vert Ax\Vert ^2, \Vert x-x^*\Vert \le \sqrt{r\alpha ^*}\} +1<\infty , \end{aligned}$$

where *L* is a Lipschitz constant of $J^{-1}.$ Define

3.4 $$\begin{aligned} \gamma _0=\displaystyle \min \dfrac{1}{2}\left\{ 1, \dfrac{kr}{M_0}\right\} \end{aligned}$$

Using the definition of $x_{n+1},$ we compute as follows:

$$\begin{aligned} \phi (x^*,x_{n+1})=&\,\, {} \phi (x^*,J^{-1}(Jx_n-\alpha _nAx_n))\\= &\,\, {} V(x^*,Jx_n-\alpha _nAx_n). \end{aligned}$$

Using Lemma 2.3, with $y^*=\alpha _nAx_n,$ we have:

$$\begin{aligned} \phi (x^*,x_{n+1})= &\,\, {} V(x^*,Jx_n-\alpha _nAx_n)\\\le &\,\, {} V(x^*,Jx_n)-2\alpha _n\langle J^{-1}(Jx_n-\alpha _nAx_n)-x^*,Ax_n-Ax^*\rangle \\= &\,\, {} \phi (x^*,x_{n})-2\alpha _n\langle x_n -x^*,Ax_n-Ax^*\rangle -2\alpha _n\langle J^{-1}(Jx_n-\alpha _nAx_n)-x_n,Ax_n\rangle \\= & \,\,{} \phi (x^*,x_{n})-2\alpha _n\langle x_n -x^*,Ax_n-Ax^*\rangle \\&\,-\,2\alpha _n\langle J^{-1}(Jx_n-\alpha _nAx_n)-J^{-1}(Jx_n),Ax_n\rangle . \end{aligned}$$

Using the strong monotonocity of *A*, Schwartz inequality and the Lipzchitz property of $J^{-1},$ we obtain

$$\begin{aligned} \phi (x^*,x_{n+1})\le &\,\, {} \phi (x^*,x_{n})-2\alpha _nk||x_n- x^*||^2 + 2\alpha _n||J^{-1}(Jx_n-\alpha _nAx_n)-J^{-1}(Jx_n)||||Ax_n\Vert \nonumber \\\le & \,\,{} \phi (x^*,x_{n})-2\alpha _nk||x_n- x^*||^2 + 2\alpha _n^2L||Ax_n\Vert ^2. \end{aligned}$$

Using Lemma 2.7, it follows that

3.5 $$\begin{aligned} \phi (x^*,x_{n+1})\le \phi (x^*,x_{n})-2\alpha _nk\phi (x^*,x_{n})+2\alpha _nk(1-c_1)||x^*||^2+\alpha _n^2M_0. \end{aligned}$$

Finally, using inequality (3.2), the definition of $\gamma _0$ (3.4), and the induction assumption, we have

$$\begin{aligned} \phi (x^*,x_{n+1})\le &\,\, {} (1-2k\alpha _n)r +\alpha _nk\dfrac{r}{2}+\alpha _nk\dfrac{r}{2}\\\le &\,\, {} \left( 1-k\alpha _n\left( 2-\dfrac{1}{2}-\dfrac{1}{2}\right) \right) r\\\le & \,\,{} (1-k\alpha _n)r. \end{aligned}$$

Therefore, $\phi (x^*,x_{n+1})\le r.$ Thus, by induction, $\phi (x^*,x_{n})\le r$ for all $n\ge 1.$ So, by inequality (2.4), $\{ x_n\}$ is bounded.

**Step 2:** We now prove that $\{ x_n\}$ converges strongly to the unique point $x^*$ of $A^{-1}(0).$ Following the same arguments as in Step 1, using the fact the sequence $\{ x_n\}$ is bounded and *A* is bounded, there exists a positive constant *M* such that

3.6 $$\begin{aligned} \phi (x^*,x_{n+1})\le \phi (x^*,x_{n})-2\alpha _nk||x_n -x^* ||^2+\alpha _n^2M. \end{aligned}$$

Therefore,

$$\begin{aligned} \phi (x^*,x_{n+1})\le \phi (x^*,x_{n})+\alpha _n^2M. \end{aligned}$$

Using the hypothesis $\displaystyle \sum _{n=0}^\infty \alpha _n^{2}< \infty$ and Lemma 2.5, it follows that $\displaystyle \lim _{n\rightarrow \infty }\phi (x^*,x_{n})$ exists. From (3.6), we have

$$\begin{aligned} \sum _{n=1}^\infty \alpha _n\Vert x_n-x^*\Vert <\infty . \end{aligned}$$

Using the fact that $\sum _{n=0}^\infty \alpha _n=\infty,$ it follows that $\displaystyle \liminf \Vert x^*- x_{n}\Vert ^2=0.$ Therefore, there exists a subsequence $\{x_{n_{k}}\}$ of $\{x_n\}$ such that $x_{n_{k}}\rightarrow x{^*}$ as $k\rightarrow \infty .$ We have

$$\begin{aligned} \phi (x^*,x_{n_{k}})=\Vert x\Vert ^2-2\langle x^*,Jx_{n_{k}}\rangle +\Vert x_{n_{k}}\Vert ^2. \end{aligned}$$

Since $\{x_n\}$ is bounded and *J* is norm-to weak$^*$ uniformly continuous on bounded subsets of *E*, it follows that $\{\phi (x^*,x_{n})\}$ has a subsequence that converges to 0. Thus, by Lemma (), $\{\phi (x^*,x_{n})\}$ converges strongly to 0. Applying Lemma(), we obtain that $\Vert x_n-x^* \Vert \rightarrow 0$ as $n\rightarrow \infty .$ This completes the proof. $\square$

### **Corollary 3.2**

*Let*$$E=L_p,$$$$1<p\le 2$$* and*$$ A: E \rightarrow E^* $$* be a bounded and strongly monotone mapping. For arbitrary*$$x_1\in E,$$* let*$$\{x_n\}$$* be the sequence defined iteratively by:*

3.7 $$\begin{aligned} x_{n+1}=J^{-1}(Jx_n-\alpha _nAx_n),\,\, n\ge 1, \end{aligned}$$

*whereJ is the normalized duality mapping fromE into*$$E^*$$* and*$$\{\alpha _n\}\subset (0,1)$$* is a real sequence satisfying the following conditions:*$$(i)\,\,\sum _{n=1}^\infty \alpha _n=\infty; $$$$(ii)\,\,\sum _{n=0}^\infty \alpha _n^{2}< \infty .$$

*Then, there exists*$\gamma _0>0$* such that if*$\alpha _n<\gamma _0,\,\,\forall \,n\ge 1$* the sequence*$\{x_n\}$* converges strongly to the unique solution of the equation*$Au=0.$

### *Proof*

Since $L_p$ spaces, $1<p\le 2$ are 2-uniformly convex Banach space with uniformly Gâteaux differentiable norm, then the proof follows from Theorem 3.1. $\square$

## Application to convex minimization problems

In this section, we study the problem of finding a minimizer of a convex function *f* defined from a real Banach space *E* to $\mathbb {R}.$

The following basic results are well known.

### **Lemma 4.1**

*Let*$$f:E\rightarrow \mathbb {R}$$* be a real-valued differentiable convex function and*$$a\in E.$$$$df:E\rightarrow E^*$$* denotes the differential map associated tof*.* Then the following hold*.

1. *The pointa is a minimizer off onE if and only if*$df(a) = 0.$
2. *Iff is bounded, thenf is locally Lipschitzian, i.e., for every*$x_0\in E$* and*$r>0,$* there exists*$\gamma >0$* such thatf* is $\gamma$*-Lipschitzian on*$B(x_0,r),$* i.e.*
  $$\begin{aligned} |f(x)-f(y)|\le \gamma \Vert x-y\Vert \,\,\forall \,x,y\in B(x_0,r). \end{aligned}$$

### **Lemma 4.2**

*LetE be normed linear space and*$f:E\rightarrow \mathbb {R}$* a real-valued differentiable convex function. Assume thatf is bounded. Then the differential map*$df: E\rightarrow E^*$* is bounded.*

### *Proof*

Let $x_0\in E$ and $r>0.$ Set $B:= B(x_0,r).$ We show that *df*(*B*) is bounded. From lemma 4.1, there exists $\gamma >0$ such that

4.1 $$\begin{aligned} |f(x)-f(y)|\le \gamma \Vert x-y\Vert \,\,\forall \,x,y\in B. \end{aligned}$$

Let $z^*\in df(B)$ and $x^*\in B$ such that $z^*=df(x^*).$ Since *B* is open, for all $u\in E,$ there exists $t>0$ such that $x^*+tu\in B.$ Using the fact that $z^*= df(x^*)$ the convexity of *f* and inequality (4.1), it follows that

$$\begin{aligned} \langle z^*, tu\rangle\le &\,\, {} f(x^*+tu)-f(x^*)\\\le & \,\,{} t\gamma \Vert u\Vert \end{aligned}$$

so that

$$\begin{aligned} \langle z^*, u\rangle \le \gamma \Vert u\Vert \, \,\forall \,u\in E. \end{aligned}$$

Therefore $\Vert z^*\Vert \le \gamma.$ Hence *df*(*B*) is bounded. $\square$

### **Definition 4.3**

A function $f:E\rightarrow \mathbb {R}$ is said to be strongly convex if there exists $\alpha >0$ such that for every $x,y\in E$ with $x\ne y$ and $\lambda \in (0,1),$ the following inequality holds:

4.2 $$\begin{aligned} f(\lambda x+ (1-\lambda ) y)\le \lambda f(x)+ (1-\lambda )f(y)-\alpha \Vert x-y\Vert ^2. \end{aligned}$$

### **Lemma 4.4**

*LetE be normed linear space and*$f:E\rightarrow \mathbb {R}$* a real-valued differentiable convex function. Assume thatf is strongly convex. Then the differential map*$df: E\rightarrow E^*$* is strongly monotone, i.e., there exists a positive constantk such that*

4.3 $$\begin{aligned} \langle df(x)-df(y),x-y\rangle \ge k\Vert x-y\Vert ^2\,\,\forall \,x,y\in E. \end{aligned}$$

We now prove the following theorem.

### **Theorem 4.5**

*LetE be a 2-uniformly convex real Banach space with uniformly Gâteaux differentiable norm and let*$f:E\rightarrow \mathbb {R}$* be a differentiable, bounded, strongly convex real-valued function which satisfies the growth condition:*$f(x)\rightarrow +\infty$* as*$\Vert x\Vert \rightarrow +\infty.$* For arbitrary*$x_1\in E,$* let*$\{x_n\}$* be the sequence defined iteratively by:*

4.4 $$\begin{aligned} x_{n+1}=J^{-1}\Big (Jx_n-\alpha _ndf(x_n)\Big ),\,\, n\ge 1, \end{aligned}$$

*whereJ is the normalized duality mapping fromE into*$$E^*$$* and*$$\{\alpha _n\}\subset (0,1)$$* is a real sequence satisfying the following conditions:*$$(i)\,\,\sum _{n=1}^\infty \alpha _n=\infty; $$$$(ii)\,\,\sum _{n=0}^\infty \alpha _n^{2}< \infty .$$* Then,f has a unique minimizer*$$a^*\in E$$* and there exists*$$\gamma _0>0$$* such that if*$$\alpha _n<\gamma _0,$$* the sequence*$$\{x_n\}$$* converges strongly to*$$a^*.$$

### *Proof*

Since *E* is reflexive, then from the growth condition, the continuity and the strict convexity of *f*, *f* has a unique minimizer $a^*$ characterized by $df (a^*)=0$ (Lemma 4.1). Finally, from Lemmas 4.2 and 4.4, the differential map $df:E\rightarrow E^*$ is bounded and strongly monotone. Therefore, the proof follows from Theorem 3.1. $\square$

## Conclusion

In this work, we proposed a new iteration scheme for the approximation of zeros of monotone mappings defined in certain Banach spaces. Our results are used to approximate minimizers of convex functions. The results obtained in this paper are important improvements of recent important results in this field.
